# Evaluations of heat action plans for reducing the health impacts of extreme heat: methodological developments (2012–2021) and remaining challenges

**DOI:** 10.1007/s00484-022-02326-x

**Published:** 2022-07-15

**Authors:** Ian J. Dwyer, Sarah J. E. Barry, Itamar Megiddo, Christopher J. White

**Affiliations:** 1 Department of Mathematics and Statistics, University of Strathclyde, Glasgow, UK; 2 Department of Management Science, University of Strathclyde, Glasgow, UK; 3 Department of Civil and Environmental Engineering, University of Strathclyde, Glasgow, UK

**Keywords:** Temperature, Extreme heat, Weather, Health, Heat plan, Evaluation, Review

## Abstract

The recent report of the Intergovernmental Panel on Climate Change is stark in its warnings about the changing climate, including future increases in the frequency and intensity of extremely hot weather. The well-established impacts of extreme heat on human health have led to widespread implementation of national and city-wide heat plans for mitigating such impacts. Evaluations of the effectiveness of some heat plans have been published, with previous reviews highlighting key methodological challenges. This article reviews methods used since and that address those challenges, so helping to set an agenda for improving evaluations of heat plans in terms of their effectiveness in reducing heat-health impacts. We examined the reviews that identified the methodological challenges and systematically searched the literature to find evaluations that had since been conducted. We found 11 evaluations. Their methods help address the key challenge of identifying study control groups and address other challenges to a limited extent. For future evaluations, we recommend: utilising recent evaluation methodologies, such as difference-in-differences quasi-experimental designs where appropriate; cross-agency working to utilise data on morbidity and confounders; adoption of a proposed universal heat index; and greater publication of evaluations. More evaluations should assess morbidity outcomes and be conducted in low- and middle-income countries. Evaluations of heat plans globally should employ robust methodologies, as demonstrated in existing studies and potentially transferrable from other fields. Publication of such evaluations will advance the field and thus help address some of the health challenges resulting from our changing climate.

## Introduction

“It is virtually certain that the frequency and intensity of hot extremes and the intensity and duration of heatwaves have increased since 1950 and will further increase in the future even if global warming is stabilized at 1.5 °C”, according to the Sixth Assessment Report (AR6) of the Intergovernmental Panel on Climate Change (IPCC) Working Group I (WGI) (Section TS-2.6 in Arias et al. 2021). In the context of the growing threat posed by extreme heat, a substantial body of epidemiological research has evidenced the impact upon human health of adversely hot weather (Cheng et al. 2019; Li et al. 2015; Song et al. 2017). Studies generally point to statistically significant impacts on cardiovascular and respiratory mortality (Arbuthnott and Hajat 2017; Cheng et al. 2019), though findings are less consistent for effects on morbidity (Martiello and Giacchi 2010), which could be due to methodological issues causing such effects to appear weaker (Astrom et al. 2011). Studies commonly report a higher relative risk for heat-related mortality and morbidity among the elderly (Li et al. 2015; Son et al. 2019). Studies examining the susceptibility of children, however, do not consistently report higher relative risk (Xu et al. 2012), and studies examining the effect of gender vary in their conclusions as to whether one group is more susceptible than the other, and if so which (Gifford et al. 2019). The extent of the health impact of hot weather can be substantial. For example, for the pan-European heatwave of August 2003, the heat-attributed mortality has been estimated at 14,802 in France alone (Kovats and Hajat 2008), and 2091 in England (Arbuthnott and Hajat 2017).

National, regional and local heat plans, aimed at mitigating the health impacts of heat extremes such as heatwaves, have become widespread and will become increasingly important as an adaptation response to climate change. Many countries now operate heat plans in which the onset of hot weather triggers a variety of interventions aimed at reducing the health impacts. Common elements of such heat plans are summarised in a guidance document published jointly by the World Health Organization (WHO) and World Meteorological Organization (WMO) (McGregor et al. 2015). Typically, heat plans consist of a Heat Warning System (HWS) which monitors a pre-defined heat metric and issues alerts of increasing severity as pre-defined thresholds for the heat metric are successively exceeded, coupled with a Heat-Health Action Plan (HHAP) which responds through public health interventions designed to mitigate possible health impacts. The proliferation of heat plans has been facilitated in Europe since 2008 (Martinez et al. 2019) by guidance on best practice, issued and updated by the WHO Regional Office for Europe and the WMO. For example, Heat Warning Systems expanded in Europe from just one country (Portugal) prior to 2001, to 16 countries by 2006 and 28 by 2009 (Casanueva et al. 2019), with many precipitated by the severe European heatwaves occurring in 2003 and 2006. To date, descriptive comparisons of European heat plans are provided by the SCORCH research programme (Vanderplanken et al. 2019b).

We note at this point that evaluations of heat plans tend to fall into two categories (our nomenclature): evaluations of *process* which examine whether the implementation of a heat plan operated as intended; and evaluations of *outcomes* which assess a heat plan’s efficacy in reducing heat-related deaths and illnesses (‘outcomes’ refers to the heath conditions resulting from extreme heat). The proliferation of heat plans and sharing of best practice underline the importance of robust methodologies for conducting evaluations of outcomes. However, given the large number of heat plans now in operation, the number of published evaluations of outcomes is low. Their apparent dearth could partly be explained by methodological challenges associated with conducting such evaluations, as identified by systematic reviews of evaluations (Bassil and Cole 2010; Boeckmann and Rohn 2014; Toloo et al. 2013). We use the term ‘methodological challenges’ to refer to difficulties in using analytical methods to estimate the effect that a particular heat plan has had on heat-related deaths and illnesses. Given the time lapse since these reviews, the proliferation of heat plans since, and the increasing periods over which many heat plans have now been operational, it is timely to review methods developed in more recent evaluations that address these methodological challenges. Such a review is the subject of this article.

Whereas evaluations of process have been comprehensively reviewed in some detail, for example Mayrhuber et al. (2018) and Vanderplanken et al. (2019a), evaluations of outcomes have not. Hence, this review is concerned only with evaluations of outcomes, and henceforth the word ‘evaluation’ in this article refers to an evaluation of outcomes.

Climate change provides a further imperative to accelerate the evaluation (and consequential improvement) of heat plans, as the occurrence of heat extremes is forecast to increase across the globe. For example, the recent IPCC assessment estimates that, for global warming of 1.5 °C, 2 °C and 4 °C, respectively, the heat event that occurred only once per 50 years during 1850–1900 is likely to occur 8.6, 13.9 and 39.2 times per 50 years (Fig SPM.6 in IPCC 2021). As well as such global warming being widely recognised as posing an increasing threat to human health (Campbell et al. 2018), including heat-related mortality (Martinez et al. 2019), so too is increased vulnerability due to an ageing population (Watts et al. 2020). In fact, the health response to increased extremes of heat is likely more complex, as several studies across the world have observed *decreasing* heat-related health impacts despite increasing temperatures (Arbuthnott and Hajat 2017; Martinez et al. 2019). This points to the important but difficult task of accounting for changes in the heat-health relationship within a given geographical region as the climate warms (Arbuthnott and Hajat 2017) and societal changes occur (such as demographic shifts, increased awareness of heat stress, new infrastructure such as increased air conditioning, and biological adaptation). These continually changing circumstances further underline the importance of robust and regular evaluations of heat plans (Martinez et al. 2019; Wu et al. 2020) to ensure that heat plans are, and continue to be, efficacious in reducing heat-related deaths and illness. Techniques used for conducting such evaluations are the topic of this review, and, to our knowledge, this is the first review with such a focus.

In this article, we explore the methodological challenges identified by the aforementioned reviews, review evaluations that have been published since 2012, and thus assess developments that have occurred and difficulties that remain. We identify the priorities going forward in terms of making best use of effective techniques that have been developed to overcome the challenges, and in terms of further work to address the remaining challenges. Our hope is that, as a timely response to IPCC’s AR6 stark warnings for a century of worsening heat extremes, this review will help accelerate best practice in the evaluation of heat plans globally, stimulate more evaluations to be conducted, encourage their publication and dissemination and ultimately lead to further optimisation and proliferation of robust heat plans, especially in regions of the world with an increasing need for them.

## Materials and methods

Our overall methodology was inspired by Levac et al. (2010) as well as the three systematic reviews referred to in (i) below. It had three components, expanded upon in this section: (i) examining the systematic reviews that previously identified methodological challenges associated with conducting evaluations of outcomes for heat plans, (ii) searching the literature for evaluations that have been conducted since those reviews and (iii) examining the methods used in the evaluations found, assessing the extent to which they overcome the challenges identified in the systematic reviews.

### Component (i)

Three systematic reviews of evaluations were examined, namely Bassil and Cole (2010), Boeckmann and Rohn (2014) and Toloo et al. (2013). Each was chosen as reviews that explicitly identify methodological challenges associated with conducting evaluations of outcomes for heat plans. Between them, these articles reviewed 18 separate evaluations of outcomes.

### Component (ii)

Three databases were searched during January 2020 with an update conducted on 4 June 2021, namely Web of Science, Scopus and PubMed. A date restriction of 2012 and later was applied to limit the search to evaluations conducted since the first of the systematic reviews studied as part of (i) above,^1^ and a language restriction of English was applied.

The search terms are provided in the Appendix. In addition, for known heat plans of major English language territories, Google and the websites of the responsible agencies were searched for evaluations of those plans (see Appendix). Grey literature was not otherwise comprehensively searched given the vast number of heat plans in operations around the globe, at national, regional and local levels, published (and possibly reviewed) in their various national languages. Thus, the emphasis of this review is on English language publications in the scientific literature.

The following exclusion criteria were then applied upon examination of titles or abstracts, in order to remove articles not relevant to the subject matter (namely methods used in evaluations of outcomes for heat plans published since those covered by the systematic reviews mentioned in (i) above):

- Not related to the effects of hot weather on health;
- Not related to heat plans;
- Concerned with long-term planning rather than (operational) heat plans;
- Not involving the evaluation of a heat plan (or component of);
- Referenced in the systematic reviews (Bassil and Cole 2010; Boeckmann and Rohn 2014; Toloo et al. 2013).
  In addition, to avoid duplication, also excluded were:
- News articles, meeting abstracts or summaries of articles already discovered.

After these exclusions were applied, publications were read fully (by the lead author) and references within each were examined for additional documents matching the selection criteria.

The above searches were designed to capture all types of evaluations, not just evaluations of outcomes, in order to capture the widest possible literature. Only then were the documents finally filtered to those that specifically included an evaluation of outcomes.

From the database searches, Web of Science (which incorporates Medline) produced 95 documents, Scopus produced 57 and PubMed produced 35, resulting in a total of 187 documents. Removing 88 duplicates and then removing another 79 by the exclusion criteria resulted in 20 documents remaining.

**Fig. 1:**
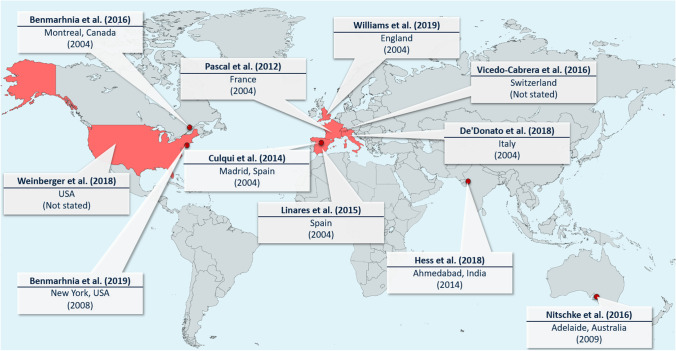
Map showing the study location of the 11 evaluations found by the literature search. Red circles show studies in specific cities; red shading shows studies across a country or region. Call-out boxes include the reference for the study, the location and the year that the evaluated heat plan (or change) was implemented

Upon reading these 20 documents in full and examining references within, 18 additional documents were identified, resulting in a total of 38 evaluations (or reviews of). Limiting these to only those that include evaluations *of outcomes* resulted in 16 documents: 11 evaluations, as detailed in Table 1 and depicted geographically in Fig. 1, plus five review articles (Boeckmann and Rohn 2014; Martinez et al. 2019; Mayrhuber et al. 2018; Toloo et al. 2013; Vanderplanken et al. 2019a).

**Table 1:**
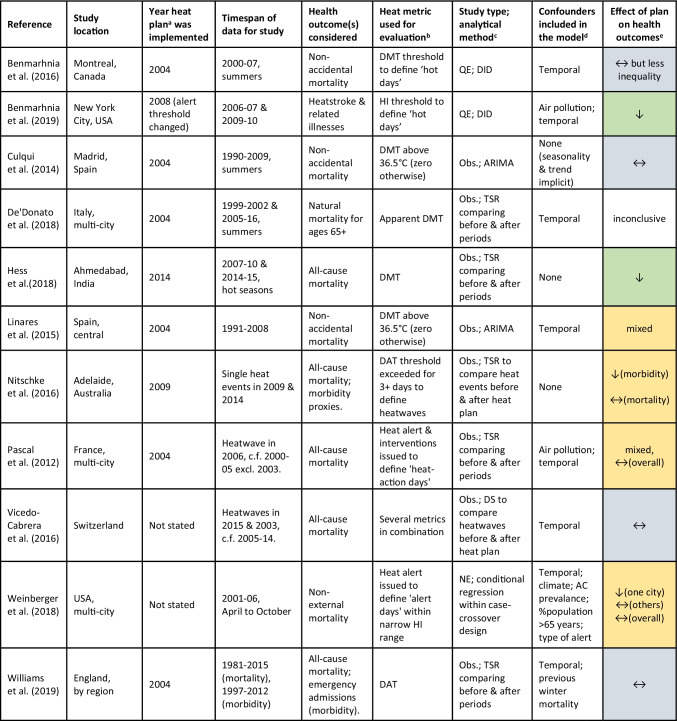
List of publications and key characteristics of the reviewed evaluations of outcomes

^a^Details of the heat plans can be mostly found within the evaluations. ^b^*DMT* daily maximum temperature, *DAT* daily average temperature, *HI* heat index. The heat metric used for evaluating the plan may differ from the metric used within the plan to issue heat alerts. ^c^Study types: *QE* quasi-experimental, *Obs*. observational study, *NE* natural experiment. Methods: *TSR* time series regression, *DID* difference-in-differences, *ARIMA* auto-regressive integrated moving average, *DS* descriptive statistics. ^d^Which temporal patterns in outcomes are adjusted for varies by study, but may include, for example, day of the week, public holidays, seasonality, long-term trends. ^e^↓ (green), health impacts reduced; ↔ (grey), health impacts unchanged; amber fill, mixed results

Regarding grey literature, the search described above did not lead to any additional documents. However, some of the heat plans found did refer to updates following some form of internal evaluation, though without providing further details or references.

It is worth noting that we discovered various literature during this search pertaining to evaluating alert thresholds used in HWSs, including, for example, Carmona et al. (2017), Nogueira and Paixao (2008), Pascal et al. (2013), Vargo et al. (2015) and Wu et al. (2020). However, whilst interesting, these did not involve evaluating the actual effect of the HWSs on health impacts and so did not constitute evaluations of outcomes and were therefore excluded.

### Component (iii)

The following data were extracted for all 11 evaluations and recorded in Table 1: geographical coverage of the heat plan under evaluation; year of heat plan implementation (or change), if specified; time period(s) of data used for the evaluation; health outcome(s) considered; the heat metric used to evaluate the heat plan; other potential explanatory factors explicitly included in the modelling (column entitled “confounders” in the table); and the conclusion in terms of the effect of the heat plan on health outcomes. Data not routinely extracted included the details of the heat plan (such as trigger thresholds and interventions) and details of the heat events occurring in the study period (such as heatwave durations); nevertheless, such data was extracted for some individual evaluations where relevant to the discussion.

For each evaluation, the study designs and modelling techniques were closely examined. Their methods were assessed against each of the challenges identified in the systematic reviews, in terms of both the extent to which they addressed each challenge and any difficulties or issues that arose as a result.

## Results

In order to recommend further work in terms of methodologies for heat plan evaluations, and thus encourage more and better evaluations to be undertaken in view of worsening heat extremes and shifting heat-health responses, we first examine the characteristics of the 11 evaluations listed in Table 1 and then compare the methodologies used against the challenges for such evaluations as identified in the three prior systematic reviews.

### Evaluation characteristics

Of the 11 evaluations (Table 1), six pertain to Europe, three to North America, and one each to India and Australia (Fig. 1). Regarding the health outcomes studied, 10 include mortality and three include morbidity, whilst two include both. Across the studies, a variety of heat metrics are used to evaluate the heat plan, some binary in nature (e.g. hot days versus non-hot days) and others continuous (e.g. daily temperature measurement). For results pertaining to mortality, only one evaluation reports an overall reduction in heat-related deaths potentially attributable to the heat plan, whilst the rest report either no reduction or, for multi-location studies, inconsistent results with no overall reduction (where calculated). For morbidity, two of the three evaluations report a reduction.

### Methodological challenges

The methodological challenges regarding evaluations of outcomes that were highlighted by the three systematic reviews (Bassil and Cole 2010; Boeckmann and Rohn 2014; Toloo et al. 2013) are collated and summarised in Box 1. The authors of these reviews note that the evaluations they examined most commonly related to mortality, compared mortality rates between time periods before and after a heat plan was implemented, and showed decreases in heat-health impacts after implementation. For example, one study for France (Fouillet et al. 2008), which compared observed excess deaths during the 2006 heatwave to excess deaths expected from the historical (pre-heat-plan) heat-health association, estimated that the national heat plan helped save 4388 lives during that heatwave. Most of the evaluations studied across these reviews (16 out of 18 separate studies) were conducted on heat plans operational in the USA and Europe (with the remaining two in China), similar to the USA-Europe predominance among the 11 evaluations found in our search.

**Table:** 

Box 1: Summary of methodological challenges in conducting evaluations of outcomes as highlighted by three systematic reviews.	
**Challenge A**: other factors affecting the heat-health association.	
*Description.* Observed reductions in health impacts for periods after heat plan implementation compared to periods beforehand could have alternative explanations not related to the introduction of the heat plan. These could include unmeasured factors that changed between the before and after periods, such as improved health care, better housing, greater public awareness about extreme heat and biological acclimatisation, which could reduce the chance of illness or death due to extreme heat and so change the heat-health association within the study period. Equally, unmeasured factors could worsen the heat-health association and obscure the effectiveness of a heat plan.	
**Challenge B**: differentiating the effects of individual interventions on reduced impacts.	
*Description.* Since heat plans typically comprise a range of interventions, often introduced simultaneously when the heat plan is implemented or updated, it is difficult to ascertain the effect of each intervention separately on any reduced health impacts.	
**Challenge C**: establishing the counterfactual scenario.	
*Description*. Difficulties arise in identifying a suitable control study group in order to ascertain the counterfactual scenario (what would have happened had the heat plan not been introduced). This is what also underlies challenges A and B. Often, the control study group is the population before implementation of the heat plan, and the experimental group is that population afterwards, thus giving rise to challenge A. An alternative could be to use as a control the population of a nearby city that is not under the new heat plan but generally subject to the same heatwaves. However, this is not a reliable control either, for example due to localised differences in the heat events as experienced, or differences in the cities’ demographics and infrastructures giving rise to different responses to the heat events. In general, contemporaneous study and reliable control groups are either not possible because geographically broad populations are usually subject to the same heat plan, or are unethical to devise (e.g. providing some but not other care home residents with increased access to fluids).	
**Challenge D:** accounting for mortality displacement.	
*Description*. Mortality displacement (sometimes referred to as the *harvesting effect*) occurs when some deaths of frail individuals are imminent anyway but are brought forward by heat stress due to the heat event. This effect may complicate the calculation of the effectiveness of response actions.	
**Challenge E**: lack of evaluations for morbidity outcomes.	
*Description*. Compared to studies involving mortality, there is a lack of equivalent studies for morbidity outcomes. This may be due to relevant morbidity data often being harder to obtain than mortality data.	
**Challenge F:** cross-study comparisons can be difficult.	
*Description*. Variation among the study designs and methods can make it difficult to undertake cross-study comparisons, such as meta-analysis.	

### Comparing the evaluations against the challenges

We assessed the 11 evaluations in Table 1 against each methodological challenge (A–F) detailed in Box 1. The detailed result of this comparison now follows and is summarised in Table 2.

**Table 2:** Summary of comparison between methodological challenges and the 11 evaluations examined. The remaining issues or difficulties under the final column were identified within the named evaluations (second column), except the two marked with an asterisk (*) which are our own observations

Challenge	Evaluation(s) addressing the challenge	Approach to addressing the challenge	Remaining issues or difficulties
Challenge A: other factors affecting the heat-health association	Benmarhnia et al. (2019), Pascal et al. (2012), Weinberger et al. (2018), Williams et al. (2019)	Measure factors when possible and include in models	Obtaining measured data is difficult for many factors
	Benmarhnia et al. (2019)	Use a short study period to reduce potential for slowly varying factors to change	Results may be sensitive to particular characteristics of a very small number of heat events in the period
Challenge B: differentiating the effects of individual interventions on health outcomes	Benmarhnia et al. (2019)	Make use of incremental changes to heat plans to evaluate in isolation	Changes need to be well isolated in time from other changes to provide enough data on extreme heat events*
Challenge C: establishing the counterfactual scenario	Weinberger et al. (2018)	Natural experiment to distinguish alerts and non-alerts for similar conditions	Results may be valid only for a narrow range of conditions
	Benmarhnia et al. (2016), Benmarhnia et al. (2019)	Difference-in-differences quasi-experimental study to create control and study groups with similar characteristics (heat events, demography, location, time period)	Challenge A remains
Challenge D: accounting for mortality displacement	De'Donato et al. (2018), Hess et al. (2018), Williams et al. (2019)	Distributed lag model, e.g. DLNM within time series regression	None
	Culqui et al. (2014), Linares et al. (2015)	Implicit in autoregressive moving average models (e.g. ARIMA)	None
Challenge E: lack of evaluations for morbidity outcomes	Benmarhnia et al. (2019), Nitschke et al. (2016), Williams et al. (2019)	Include morbidity or morbidity proxies in evaluation	Morbidity data often harder to obtain
Challenge F: cross-study comparisons can be difficult	None	Greater consistency between studies, e.g. use of universal heat index (Nairn et al. 2018)	May reduce diversity of approaches*

#### Challenge A: other factors affecting the heat-health association

Many of the reviewed evaluations that compare health outcomes between periods before and periods after heat plan implementation acknowledge other possible explanatory factors, not measured or explicitly controlled for in the analysis, which could have changed within the study period and affected the heat-health relationship. Factors cited included air quality (Culqui et al. 2014), awareness within the population of heat effects (Hess et al. 2018), infrastructure characteristics such as the prevalence of air conditioning (De'Donato et al. 2018) and living conditions (Hess et al. 2018).

In principle, such factors can be accounted for in quantitative analysis if appropriate observed time-series data is available (in which case they become measured rather than unmeasured factors), as was done for the evaluation of the French heat plan (Pascal et al. 2012) by including a term for air pollution in a regression model. Whilst obtaining the necessary data for such factors may be relatively straightforward for variables like air pollution, it can be much more difficult for other factors. To help address this challenge, the evaluation for New York City (Benmarhnia et al. 2019) deliberately chose a short study period (2006–2010) to limit the potential for influence from slowly varying unmeasured factors. However, the authors for the evaluation for Spain (Linares et al. 2015) used a much longer study period (1991–2008), arguing that the results from short-period studies that contain a small number of heat events can be too sensitive to the particular characteristics of those events (intensity, duration, time of year). An alternative method to account for other explanatory factors, however, is to undertake an experimental or quasi-experimental study (see challenge C below).

#### Challenge B: differentiating the effects of individual interventions on health outcomes

This challenge is acknowledged in several of the reviewed evaluations (De'Donato et al. 2018; Pascal et al. 2012; Vicedo-Cabrera et al. 2016). For most heat plans, once a heat alert is issued, different interventions are simultaneously initiated, so attributing any reduction in health impacts to individual actions is therefore problematic. The study for New York (Benmarhnia et al. 2019) utilised a 2008 change in the heat index threshold used for issuing city-wide heat alerts and examined the effect of that single change on the daily rates of heatstroke and related illnesses for over 65-year-olds. By comparing the two, 2-year periods either side of the change, whilst adjusting for air pollution and temporal patterns in the health outcome, they found a small but statistically significant reduction in heat illnesses. It was the only evaluation we reviewed that addressed this challenge, albeit evaluating the change in the heat plan rather than its very existence. Commenting on this study, Ebi (2019) suggests that studies of incremental changes to heat plans may offer a promising way forward. Nevertheless, the approach is not without difficulties, as a single incremental change may be required to be isolated from other changes several years either side to afford enough data on extreme heat events to achieve sufficient statistical power within the analysis. Also, the approach can only evaluate *changes* to interventions.

#### Challenge C: establishing the counterfactual scenario

In the absence of practical controlled experiments, evaluations of heat plans are conducted as observational rather than experimental studies. It can therefore be difficult to establish the counterfactual scenario (Rothman et al. 2012).

Three of the evaluations, however, did attempt to address this problem. The USA study (Weinberger et al. 2018) evaluated the effectiveness of heat alerts, issued by the USA’s national alert system, on reducing mortality across 20 US cities over the period 2001–2006 (April to October only). Alerts are issued if the calculated heat index exceeds a certain threshold. Their study design exploited the differences between actual (observed) weather and forecasted weather: some heat events observed slightly below the heat index threshold generated false heat alerts because they were wrongly forecast to exceed the threshold, whilst others slightly above the threshold resulted in false non-alerts as they were wrongly forecast not to exceed the threshold. For each city, then, a set of days spread across the study period can be identified that have similar heat indexes, but with some resulting in a heat alert and others not. The study thus establishes the counterfactual scenario based upon the ‘control’ group of days for which no alert resulted. This is a ‘natural experiment’—one that defines the experimental and control groups according to observed factors that occurred naturally. In this study, a case-crossover design with logistic regression was then used to estimate incident rate ratios (IRRs) for the occurrence of death for the alert days versus the non-alert days. (The case-crossover design is increasingly popular for observational studies of acute outcomes due to short-term exposures (Lu and Zeger 2007) as a way of controlling for both time-invariant individual characteristics such as sex, age and health, and temporal confounders such as seasonality and trend in outcomes.) Thus, a control group is established that is not separated from the experimental group in either time or space or key demographics, which thus helps avert influence from a number of possible unmeasured factors on the results. However, the results are not generalizable beyond the narrow range of values of the heat index that were used to generate the alert days and non-alert days. The study design addresses a different question to evaluations based on ‘before’ and ‘after’ heat plan introduction, in that it asks whether carrying out an intervention (issuing a heat alert in this case) reduces excess mortality, rather than asking whether having a heat plan is associated with reduced heat-health impacts. The study results show a protective effect of heat alerts for only one of the 20 cities (Philadelphia), and no overall protective effect (as estimated from a random effects meta-analysis). The authors also performed a meta-regression to examine variation in the protective effect by city characteristics (such as proportion of the population over 65 and prevalence of air conditioning), finding none.

Two of the evaluations—for Montreal (Benmarhnia et al. 2016) and for New York (Benmarhnia et al. 2019)—use a “quasi-experimental” design to estimate the causal effect of a heat plan on mortality and morbidity respectively. For the Montreal evaluation, instead of comparing (non-accidental) mortality directly between periods before and after heat plan implementation, the authors used a difference-in-differences (DID) analysis which estimates the difference between (a) the difference in mortality before compared to after periods, on *hot days* only, and (b) the same but for *non-hot* days only. (A ‘hot day’ was one in which the HWS alert heat threshold was reached, whether before or after heat plan implementation.) This DID quantity represents the reduction in mortality on hot days that are due to the heat plan and is estimated through Poisson time series regression. The rationale being that many non-hot days in the location of interest can thereby be utilised in order to establish a control group, as opposed to, for example, using relatively few hot days for a control group in a different location without the heat plan (which also introduces differences in population characteristics between the groups). However, this is still in essence a before versus after comparison (see challenge A), so the estimate of the effect of the heat plan is only valid if no other unmeasured factors affected the heat-health relationship during the study period, and for this reason, the authors chose a short study period. They found non-significant overall mortality reduction (5% significance level), but did find statistically significant reductions for the elderly and lower socio-economic groups and thus provided evidence that the heat plan reduced *inequalities* in heat-related mortality. The New York study used the same (DID) approach to find a modest reduction in heat-related morbidity in the elderly after the lowering of the threshold used for issuing heat alerts. Whilst this approach does not solve the problem in challenge A above regarding unmeasured confounders in before-versus-after comparisons, it does seek to establish the counterfactual scenario through a control group which at least has experienced the same climate, has the same demographics, and is studied over the same time span, as the experimental group.

#### Challenge D: accounting for mortality displacement

Short-term mortality displacement can be investigated in a time series modelling approach by incorporating multiple time lags for the heat variable into the model through a distributed lag model (Armstrong 2006; Bhaskaran et al. 2013). A displacement effect can be detected from the difference in risk at longer lags. Such models are also used to investigate short-term delays (lags) in the effect of the high temperatures on the health outcome of interest. For example, the evaluation of Italy’s heat plan (De'Donato et al. 2018) employed a well-established version of such models known as a distributed lag nonlinear model (DLNM). Comparing, separately for 23 cities across Italy, natural mortality for 65-year-olds before and after the introduction of a national heat plan, the DLNM considered lags of 0 to 7 days with no displacement effect reported. The study for Ahmedabad (Hess et al. 2018) also used a DLNM which included lags 0–5 days. The study for England (Williams et al. 2019) included a lag model and an investigation of seasonal-scale mortality displacement by calculating, for separate English regions, the correlation between annual winter deaths and deaths in the following summer (after de-trending both time series), with no statistically significant correlations found.

#### Challenge E: lack of evaluations for morbidity outcomes

Of the 18 separate evaluations of outcomes covered by the three aforementioned reviews (Bassil and Cole 2010; Boeckmann and Rohn 2014; Toloo et al. 2013), only three (17%) examined morbidity. By comparison, 27% (three out of 11) covered by this review examined morbidity. For New York, Benmarhnia et al. (2019) found that a change in the alert threshold for the heat plan produced a minor reduction in heatstroke and heat-related illnesses. For Adelaide, Nitschke et al. (2016) reported that the heat plan resulted in a reduction in morbidity as measured by ambulance call-outs and presentations to emergency facilities relating to specific heat-related conditions. On the other hand, Williams et al. (2019) found no reduction in emergency admissions attributable to England’s heat plan. This might suggest an upturn in morbidity studies, perhaps due to increased availability of relevant data or a greater recognition of the need for such studies, though it is a small sample and still the proportion of morbidity studies remains low.

#### Challenge F: cross-study comparisons can be difficult

As summarised in Table 1, the 11 evaluations that we have reviewed exhibit a variety of study designs (observational, quasi-experimental), analytical methods (DID, ARIMA, time series regression, case-crossover) and health outcomes (all-cause mortality, non-accidental mortality and various morbidity measures). The general lack of a standard approach continues to be lamented (Bittner et al. 2014; Vaidyanathan et al. 2019). The absence of a commonly agreed heat metric for issuing heat alerts is often noted (Casanueva et al. 2019).

Australia’s Bureau of Meteorology (BoM) and Commonwealth Scientific and Industrial Research Organisation (CSIRO) are trying to address the latter point by proposing a universal heat index (Nairn and Fawcett 2013) which appears promising in its ability to predict health impacts (Nairn et al. 2018). A location-specific measure of heatwave intensity accounts for both short- and long-term local temperature anomalies and is converted to a (non-dimensional) severity index that is comparable between locations and events. The severity index correlates well with health impacts, and statistics for the index (e.g. peak value) can correctly rank heat events according to their health impacts (especially for mortality). None of the 11 evaluations we examined used this heat metric.

## Discussion

In this study, we collated evaluations of heat-health action plans that assessed their effectiveness in reducing the adverse health outcomes of hot weather extremes. We examined 11 evaluations (Table 1) appearing since the publication of three review articles that identified a number of methodological challenges associated with conducting such evaluations (Box 1). We assessed the 11 evaluations against the methodological challenges to identify methods that address the challenges (Table 2). In this section, we review the key findings and explore the priorities for further work that could help improve and encourage further evaluations of outcomes.

Challenge C (establishing the counterfactual scenario) is perhaps the most fundamental of the six challenges evaluated as it pertains to the key task of establishing the baseline (i.e. what would have happened in terms of heat-health impacts had the heat plan not been implemented) against which to assess the effectiveness of the implemented heat plan (or part of one). As detailed in the results section and summarised in Table 2, the three North American studies use some promising techniques that address this challenge by devising natural experiments or quasi-experimental studies that compare populations with greater similarities (in time, demographics and location) than generally found in purely observational studies. One approach, for example, involved the DID methodology, which is commonly used in disciplines such as econometrics (Angrist and Pischke 2008).

Recommendation 1. Future evaluators should examine the natural experiment and quasi-experimental methodologies in the well-devised studies of Benmarhnia et al. (2016), Benmarhnia et al. (2019) and Weinberger et al. (2018) for opportunities to maximise the robustness of their own evaluations. Furthermore, other fields of study, such as economics or assessments of public interventions to curb the spread of SARS-CoV-2, should be examined for state-of-the-art applications of methodologies that are transferrable to evaluations of outcomes for heat plans.

One issue not addressed by the DID methodology is challenge A (other factors affecting the heat-health association, such as changes in air pollution, prevalence of air conditioning or demographics). For *measured* factors, time series regression models can readily include them and thus account for them. However, only four of the 11 evaluations explicitly included other measured factors (besides adjusting for temporal patterns such as seasonality), two of which only accounted for air pollution (see Table 1). This may be understandable given that air pollution data is relatively easy to obtain compared to data for other factors like infrastructure developments or improvements in underlying population health. Nevertheless, effective multi-agency working must surely be able to result in the wider availability of relevant data given the increasing emphasis nowadays on measurement and data in most sectors. An additional approach used in some evaluations to avoid difficulties in collecting data on slowly varying factors like underlying population health is to choose a short study period to limit the extent of such variations; this having the potential disadvantage of resulting in a limited number and range of heat events during the study period on which to base comparisons.

Recommendation 2. We urge that every effort is made to obtain data on the most likely alternative explanatory factors, whether directly measured or using proxy indicators, through appropriate partnership working when designing and evaluating heat plans. Judgement is therefore required to balance the collection of data on additional factors and using a longer study period, against the difficulty of obtaining such data and the likely extent of changes in slowly-varying factors.

None of the 11 evaluations attempted to differentiate the effects of individual interventions on outcomes (challenge B), though the New York study examined the effect of a single incremental change to a heat plan. Evaluating individual interventions by utilising incremental changes in heat plans requires incremental changes to have occurred and be sufficiently isolated in time to allow a suitably long study period with sufficient heat extremes occurring. This challenge thus remains a difficulty, with no demonstrations of how to tackle it for heat plan evaluation. Nevertheless, many heat plans are now in operation, and some incremental changes involving single interventions may well have occurred.

Recommendation 3. We encourage maximum utilisation of any incremental changes to heat plans that occur, through evaluations of their effectiveness using before versus after studies. Furthermore, though no examples are available in the heat plan literature, other fields of study may offer methodologies for differentiating between interventions. Recent scientific literature studying the effectiveness of multiple interventions for curbing the spread of SARS-CoV-2 may offer insights (Dehning et al. 2020; Haug et al. 2020).

Accounting for mortality displacement and response time lags when estimating heat-health responses (challenge D) can be readily done within a time series regression framework through the use of a distributed lag nonlinear model (DLNM), for example, as demonstrated in three of the 11 evaluations (Table 2). We see no reason that the other evaluations using time series regression could not have incorporated such techniques. Two other evaluations use ARIMA time series modelling, which implicitly accounts for these effects through the suitable choice of relevant model parameters.

Recommendation 4. Whenever mortality displacement and response time lag could possibly affect the estimation of health impacts, we encourage using techniques that account for these, such as DLNM in time-series regression or ARIMA models. We would like to see the application of distributed lag models demonstrated in a DID framework and case-crossover design.

The apparent upturn in the relative number of evaluations dealing with morbidity (challenge E) is encouraging, though still represents a minority (3 of 11) compared to mortality studies. Warning levels are often determined based on health outcomes, with increasing levels generally pertaining to increasing health risks (Vanderplanken et al. 2019b). Lower warning levels may therefore be geared more towards mitigating morbidity than mortality, so the effectiveness of heat plans in reducing morbidity should be equally evaluated. Whilst morbidity (or proxy) data may be generally harder to obtain than mortality data, we make a similar recommendation as for challenge A.

Recommendation 5. Every effort should be made through partnership working to ensure that relevant morbidity data can be collected for the purpose of conducting a comprehensive evaluation of outcomes.

The lamented problem of difficulties in comparing or pooling studies due to variability in study designs and methods (challenge F) was noted above. Whilst we sympathise with this challenge, we stop short of recommending uniformity in evaluation design. Diversity in the 11 evaluations we have examined has given rise to a range of approaches for tackling different methodological challenges. We propose that the emphasis should be on robust evaluation, whatever underlying study designs are employed, with respect to some of the key challenges described above (A–E). If evaluations are individually more robust, rendering greater confidence in their results and conclusions, then these will be more meaningfully compared (than, say, a set of less robust evaluations that all use the same design). We have noticed, however, the very varied heat metrics that are used in evaluations (Table 1), which do not necessarily correspond to the heat metrics used to design each heat plan and issue heat alerts.

Recommendation 6. In light of the promising literature regarding the ability of the universal heat index developed by BoM and CSIRO to forecast health impacts (Nairn and Fawcett 2013; Nairn et al. 2018), we recommend that this heat index is strongly considered for both the design and evaluation of heat plans.

Despite the increased emergence of heat plans and their increasing longevity, the results of this search suggest few evaluations of outcomes have been published in the peer-reviewed scientific literature over the last five or so years. This has also been noted by others (Martinez et al. 2019). Although our search was limited to the English language, a multi-language search for evaluations (Vanderplanken et al. 2019a) did not reveal many either; and though we did not systematically scour the grey literature, anecdotally we observed that ‘in-house’ evaluations of heat plans appear to have been conducted as part of an annual or periodic internal review processes, but not published or made public.

Recommendation 7. We encourage publication and dissemination of heat plan evaluations, for the benefit of shared best practice.

A few additional observations are worth noting. First, only one of the 11 evaluations studied, namely for Ahmedabad in India (Hess et al. 2018), is outside the wealthiest group of countries. The 18 unique evaluations found by the prior systematic reviews were similarly dominated by richer nations. This echoes concerns expressed elsewhere over the lack of studies in countries that are particularly prone to heat and have the least capacity to respond (Campbell et al. 2018; Green et al. 2019; Romero-Lankao et al. 2012). As noted by the recent IPCC report (B.2.3 in IPCC 2021), some mid-latitude and semi-arid regions are projected to see the highest increase in the temperature of the hottest days. Second, whilst the 16 mortality studies previously reviewed predominantly report reductions in heat-mortality associations after heat plans were introduced, only one out of the 11 mortality studies listed in Table 1 does so. However, it is possible that earlier studies suffered more reporting bias (Lian et al. 2015)—that is, a higher likelihood of publication of positive rather than negative or inconclusive results. Finally, we commend the study for Montreal (Benmarhnia et al. 2016) for explicitly examining the effects of the heat plan on the key matter of health inequalities.

Challenges (A–F) all sit within a broader set of challenges facing the implementation and evaluation of heat plans, as expressed in some recent articles (Martinez et al. 2019; Mayrhuber et al. 2018). However, to our knowledge, this review is the first that focuses on *evaluations of outcomes* and elucidates their methodologies to expressly examine their utility for addressing specific, key methodological challenges highlighted by prior literature.

## Conclusions

The increasing importance of heat plans for mitigating the health impacts of heat extremes is clear. They need to be effective, and as such must be evaluated and re-evaluated as the climate changes and heat-health associations modify. Methodologies for evaluating their effectiveness in mitigating heat-related deaths and illness need to be robust: addressing morbidity as well as mortality; attributing mitigation to the heat plan; differentiating, where possible, the effectiveness of different interventions; accounting for other socio-economic factors affecting the heat-health association as well as various temporal effects; and conducted on heat plans globally, including for low- and middle-income countries. Best practice needs to be elucidated and shared through prominent publication. This review found surprisingly few recent evaluations in the scientific literature, despite the rapid proliferation of operational heat plans. Nevertheless, some techniques used in those evaluations have addressed previously identified methodological challenges in conducting such evaluations, though some difficulties persist. We made seven recommendations for future work, aimed at facilitating robust evaluations of heat plans globally and encouraging their publication as a timely response to a rapidly warming climate.

## Data Availability

Not applicable.

## Appendix

The following search terms were used for the published articles.

Article Title: (heat* OR “extreme heat” OR “extreme temperature” OR “high temperature” OR “extreme temperatures” OR “high temperatures” OR “hot weather”) AND (early-warning* OR warning* OR plan OR plans OR hws OR hhws OR hhap OR alert OR intervention*) AND (assess* OR evaluat* OR apprais* OR validat* OR effective* or efficacy OR review OR compar* OR impact* OR survey*).

For the following known heat plans, the stated agency websites were searched for evaluations (location - name of plan - agency website examined):

Queensland, Australia - Queensland Health Heatwave Management Sub-Plan - https://www.publications.qld.gov.au/

Toronto, Canada - City of Toronto Hot Weather Response Plan / Framework - https://www.toronto.ca/

Canada - (national) Heat Alert and Response Systems (HARS) - https://www.canada.ca/en/health-canada.html

Victoria, Australia - Heat health plan for Victoria - https://www2.health.vic.gov.au/

South Australia - Extreme heat strategy - https://www.ses.sa.gov.au/site/home.jsp

New Zealand - Heat Health Plans - https://www.health.govt.nz/

## Footnotes

1 The year of the first of the three systematic reviews (2012) rather than the last (2014) was chosen out of caution as it was noticed that at least one relevant evaluation published in 2012 was not captured by any of the three systematic reviews. We also recognised that evaluations may have been published between the first and last systematic reviews whilst the latter two were in the process of publication.
